# The Endoplasmic Reticulum Chaperone Calnexin Is a NADPH Oxidase NOX4 Interacting Protein*[Fn FN2]

**DOI:** 10.1074/jbc.M115.710772

**Published:** 2016-02-09

**Authors:** Kim-Kristin Prior, Ilka Wittig, Matthias S. Leisegang, Jody Groenendyk, Norbert Weissmann, Marek Michalak, Pidder Jansen-Dürr, Ajay M. Shah, Ralf P. Brandes

**Affiliations:** From the ‡ Institut für Kardiovaskuläre Physiologie, Goethe-Universität, Frankfurt am Main, 60590 Germany,; the ¶ Functional Proteomics, SFB 815 Core Unit, Goethe-Universität, 60590 Frankfurt am Main, Germany,; the ‖ Cluster of Excellence “Macromolecular Complexes,” Goethe-Universität, 60590 Frankfurt am Main, Germany,; the ** Department of Biochemistry, University of Alberta, Edmonton, Alberta T6G 2H7, Canada,; the ‡‡ Excellence Cluster Cardio-Pulmonary System, Justus-Liebig-University Member of the German Center for Lung Research (DZL), 60590 Giessen, Germany,; the §§ Institute for Biomedical Ageing Research and Center for Molecular Biosciences Innsbruck (CMBI), Universität Innsbruck, 6020 Insbruk, Austria,; the ¶¶ King's College London British Heart Foundation Centre, Cardiovascular Division, London WC2R 2LS, United Kingdom, and; the § German Center for Cardiovascular Research (DZHK), Partner site RheinMain, 60590 Frankfurt am Main, Germany

**Keywords:** mass spectrometry (MS), NADPH oxidase, protein-protein interaction, proteomics, reactive oxygen species (ROS), blue native PAGE

## Abstract

Within the family of NADPH oxidases, NOX4 is unique as it is predominantly localized in the endoplasmic reticulum, has constitutive activity, and generates hydrogen peroxide (H_2_O_2_). We hypothesize that these features are consequences of a so far unidentified NOX4-interacting protein. Two-dimensional blue native (BN) electrophorese combined with SDS-PAGE yielded NOX4 to reside in macromolecular complexes. Interacting proteins were screened by quantitative SILAC (stable isotope labeling of amino acids in cell culture) co-immunoprecipitation (Co-IP) in HEK293 cells stably overexpressing NOX4. By this technique, several interacting proteins were identified with calnexin showing the most robust interaction. Calnexin also resided in NOX4-containing complexes as demonstrated by complexome profiling from BN-PAGE. The calnexin NOX4 interaction could be confirmed by reverse Co-IP and proximity ligation assay, whereas NOX1, NOX2, or NOX5 did not interact with calnexin. Calnexin deficiency as studied in mouse embryonic fibroblasts from calnexin^−/−^ mice or in response to calnexin shRNA reduced cellular NOX4 protein expression and reactive oxygen species formation. Our results suggest that endogenous NOX4 forms macromolecular complexes with calnexin, which are needed for the proper maturation, processing, and function of NOX4 in the endoplasmic reticulum.

## Introduction

NADPH oxidases of the NOX family are important sources of reactive oxygen species (ROS).^4^ In mammals, seven homologues are expressed that can be grouped according to their mode of activation. NOX1, NOX2, and NOX3 are mainly activated by protein-protein interaction with cytosolic proteins, NOX5, DUOX1, and DUOX2 (Dual oxidase 1 and 2) are calcium-dependent, whereas NOX4 is constitutively active. The lack of activity control for NOX4 is enigmatic given the potential harmful effects of excessive ROS production. It is therefore believed that NOX4-dependent ROS production is largely controlled through the abundance of the enzyme (1). Indeed, certain conditions like hypoxia or the stimulation with transforming growth factor β1 (TGF-β1) are known to strongly induce NOX4 in fibroblasts or epithelial cells (2, 3). On the other hand, fairly high mRNA levels for *Nox*4 are detected in almost all differentiated tissues, but protein expression by Western blot analysis is readily detectable only in a few tissues, such as in the kidney, lung, and also the endothelium (4–6). It can be speculated that NOX4 is not only regulated on the mRNA level itself but also by additional posttranslational mechanisms. Furthermore, a potential explanation for missing oxidative stress but constitutive expressed NOX4 is that NOX4 produces predominantly H_2_O_2_ rather than O_2_^˙̄^ as do NOX1, NOX2, NOX3, and NOX5 (7–9) and that NOX4 might be located at a specific site in the cell. The latter aspect has long been debated, with numerous subcellular locations such as the plasma membrane, nucleus, mitochondria, and endoplasmic reticulum (ER) being associated with NOX4 (10, 11). This controversy is obviously fueled by the difficulties in detecting the NOX4 protein, by problems with antibody specificity and the potential that NOX4 might shuttle between compartments. It is understandable that the ER is a site of NOX4 expression as being a membrane protein, NOX4 has to be synthesized and folded in the ER. Other locations, like the nucleus (12, 13) are less easy to explain and expression at the plasma membrane might be restricted to certain cell lines or occurs only in response to certain stimuli (14).

Given the differences in NOX4 protein expression and the different locations as well as a certain conceptual desire to identify mechanisms of direct activity control, NOX4 interacting proteins could be postulated. For the DUOX enzymes, for example, maturating factors are required to achieve sufficient expression and activity; on the other hand the interaction with p22*^phox^* is required for NOX2 to stabilize the protein and to allow maturation (15). Some observations suggest an interaction of NOX4 with p22*^phox^* (16, 9) although the exact site where p22*^phox^* binds to Nox4 differs from that of NOX1 and NOX2 (17). Moreover, through the search for p22*^phox^* interacting proteins by yeast 2-hybrid screening, polymerase δ interacting protein 2 (POLDIP II) has been found to control NOX4 activity (18) and also protein-disulfide isomerase is believed to interact with the enzyme (19).

On the basis of these findings we hypothesized that additional so far unidentified NOX4 interacting proteins may exist. We therefore employed a NOX4 overexpression system to screen for potential interactors, which were subsequently validated in a desire to determine the functional importance in primary cells.

## Experimental Procedures

### 

#### 

##### Cell Culture

Human embryonic kidney 293 cells (HEK293) were obtained from ATCC (Manassas, VA) and cultured in modified Eagle's medium (MEM, Gibco) supplemented with fetal calf serum (FCS; 8%), non-essential amino acids (0.1 mm), sodium pyruvate (1 mm), and gentamycin (50 μg/ml) in a humidified atmosphere (5% CO_2_, 37 °C).

HEK293T/17 cells were purchased from ATCC (Manassas, VA). Cells were cultured in Dulbecco's modified Eagle's medium (DMEM), high glucose, GlutaMAX (Gibco), supplemented with 8% FCS, penicillin (50 units/ml), and streptomycin (50 μg/ml) in a humidified atmosphere of 5% CO_2_ at 37 °C.

##### Isolation of Mouse Embryonic Fibroblasts (MEF)

MEFs were isolated from wild type (wt) and calnexin-knock-out (*Canx*^−/−^) mice as described before (20) and provided by one of the co-authors (M.M.) (20). Cells were cultured in DMEM, high glucose, GlutaMAX (Gibco) supplemented with 8% FCS, penicillin/streptomycin in a humidified atmosphere (5% CO_2_, 37 °C).

##### Isolation of Podocytes from Kidney

Primary podocytes were isolated as described (21, 22). Briefly, transcardiac perfusion was performed with 8 × 10^7^ Dynabeads M450, tosyl-activated (Invitrogen), and suspended in podocyte growth medium (RPMI 1640 (Gibco), 8% FCS, 5 mm HEPES, 0.1% non-essential amino acids, 0.1 mm sodium pyruvate, 0.01 mg/ml of insulin/transferrin/sodium seletine solution, penicillin/streptomycin). Mouse kidneys were minced into 1-mm^3^ pieces. These were digested with 1 mg/ml of collagenase A (Roche Applied Science) in podocyte growth medium (37 °C, 30 min) and afterward pressed twice through two 100-μm cell strainers using a flattened pestle. Cell suspensions were centrifuged at 200 × *g* for 5 min and the cell pellet was resuspended in 5 ml of podocyte growth medium. Glomeruli containing Dynabeads were isolated by a magnetic particle concentrator and washed with podocyte growth medium. Eventually glomeruli of three each of wt or *Nox4*^−/−^ mice were combined and cultured in podocyte medium in a humidified atmosphere (5% CO_2_, 37 °C).

##### Stable Isotope Labeling of Amino Acids in Cell Culture (SILAC)

For labeling of HEK293 cells with isotopic amino acids, cells were grown in DMEM without lysine, arginine, and glutamine (280001200, SILANTES), supplemented with 8% dialyzed FCS, l-glutamine (2 mm), l-proline (200 mg/liter), glucose (4.5 g/liter), sodium pyruvate (1 mm), 1% penicillin/streptomycin, and 100 mg/liter of [^13^C]arginine and [^13^C,^15^N]lysine (heavy; H*) or [^12^C,^14^N]arginine and [^12^C,^14^N]lysine (light; L) in a humidified atmosphere (5% CO_2_, 37 °C) to ensure total incorporation of labeled amino acids. Heavy amino acid labeling was checked after 14 days cell culture.

##### Transfection of NOX Constructs

Transient transfection of HEK293 cells was performed using Lipofectamine 2000 (Invitrogen) and 0.75 μg/ml of DNA according to the manufacturer's instructions. HEK293 cells stably expressing NOX1 (NOX1-HEK293) or NOX4 (NOX4-HEK293) were generated as described before using lentiviral transfection and selection (10).

##### Small Hairpin RNA (shRNA) Transduction

For lentiviral knockdown, two different shRNA were purchased from Sigma (sh1, TRCN0000278234; sh2, TRCN0000278235). As controls scrambled shRNA (Addgene number 1864) and GFP shRNA (Addgene number 30323) from David Sabatini were used. The controls as well as the packing plasmid (Addgene number 12260) and envelope plasmid (Addgene number 12259) were a gift from Didier Trono. Transfection and transduction were performed as described in the manufacturer's protocol. HEK293 cells were selected for at least 7 days with 2 μg/ml of puromycin after transduction.

##### Determination of ROS Production

ROS production was measured as described before (23). As probe, luminol (100 μm, Sigma) catalyzed by horseradish peroxidase (HRP, 1 units/ml, Sigma) or L012 (250 μm, Wako) was used and measured in a Berthold 6-channel luminometer (LB9505, Berthold, Wildbad, Germany) or TriStar^2^ Multimode Reader (LB942, Berthold, Wildbad, Germany).

H_2_O_2_ formation was also measured with the Amplex Red assay (50 μm, Invitrogen; HRP, 2 units/ml, Sigma) as previously described (23). In some experiments cells were stimulated with TGF-β1 (10 ng/ml, Peprotech) for 24 h to induce NOX4. To block NOX activity, diphenylene iodonium (10 μm, Sigma) was added for 1 h. Respective fluorescence was determined in a microplate fluorimeter (excitation 530 nm, emission 590 nm). Protein amount was determined by Bradford protein assay and used for Western blot analysis.

##### Membrane Preparation by Differential Centrifugation

HEK293 cells were mechanically homogenized in homogenization (HM) buffer (250 mm sucrose, 10 mm Tris/HCl, pH 7.4, 1 mm EDTA, 2 mm orthovanadate, 10 nm okadaic acid, protein-inhibitor mixture, 40 μg/ml of phenylmethylsulfonyl fluoride (PMSF)) using a pre-cooled motor-driven glass/Teflon Potter-Elvehjem homogenizer at 2000 rpm and 40 strokes. Subcellular components were separated by differential centrifugation at 4 °C at 3,000 × *g* for 10 min, 9,000 × *g* for 20 min, and 15,000 × *g* for 30 min to gain a pellet and supernatant. Pellets were resuspended in 1 ml of HM buffer containing protease inhibitors and used for the protein amount determination by Bradford assay and subsequently for Western blot analysis or membranes were spun down at 100,000 × *g* for 15 min (4 °C) and used for blue native electrophoresis.

##### Co-immunoprecipitation (Co-IP)

For immunoprecipitation of HSPA5 (Santa Cruz, number sc-1051), CANX (Merck Millipore, number MAB3126), or GFP (Roche, number 11814460001) HEK293 cells were lysed in Tris/HCl, pH 7.5, buffered with 1% Triton or digitonin containing protease inhibitors as described above. Supernatant was incubated with appropriate antibody (2 μg) for at least 90 min at 4 °C followed by incubation for an additional 90 min with Protein A/G-Sepharose beads (GE Healthcare). Beads were washed with lysis buffer three times, incubated for 5 min at 95 °C with Laemmli buffer, and subjected to SDS-PAGE and Western blot analysis.

For quantitative SILAC-based NOX4-Co-IP (qCo-IP) HEK293 cells were grown in normal or heavy labeled SILAC medium (100 mg/liter of [^13^C]arginine/[^13^C,^15^N]lysine or [^12^C,^14^N]arginine/[^12^C,^14^N]lysine), lysed with either Tris/HCl, pH 7.5, buffered 1% digitonin or for total membrane preparation the cells were harvested in HM buffer, nuclei were removed (3,000 × *g* for 10 min), and 100,000 × *g* total membrane (20 min, 4 °C) pellets were solubilized with digitonin (6 g/g of protein) in solubilization buffer A (50 mm NaCl, 50 mm imidazole/HCl, pH 7.0, 2 mm 6-aminohexanoic acid, 1 mm EDTA). Samples were used for NOX4 qCo-IP with magnetic protein G bead (Invitrogen) analogs to normal Co-IP as described. Additionally, the NOX4 antibody (24) was covalently linked to the beads with 20 mm dimethyl pimelimidate dihydrochloride (Sigma) in 0.2 m triethanolamine, pH 8.2 (Sigma). Light and heavy labeled samples were combined and analyzed in SDS-PAGE and Western blot analysis as well as mass spectrometry.

##### Isolation of Macromolecular Complexes by Blue Native Gels

15,000 × *g* membrane pellets from the differential centrifugation containing 200 μg of protein were resuspended in 20 μl of buffer A (50 mm NaCl, 50 mm imidazole, pH 7, 1 mm EDTA, 2 mm aminocaproic acid) and solubilized with detergent/protein ratios of 6 or 12 g/g with digitonin (Serva), 2.5 or 5 g/g of *n*-dodecyl β-d-maltopyranoside (Glycon, Luckenwalde), and 3 or 6 g/g of Triton X-100 (Sigma). Samples were centrifuged for 20 min at 22,000 × *g* and the supernantant was supplemented with 2.5 μl of 50% glycerol and 3 μl of Coomassie dye solution (5% Serva Blue G250, 500 mm aminocaproic acid). Samples were loaded onto 3 to 18% acrylamide gradient gels following blue native-polyacrylamide gel electrophoresis (BN-PAGE) as described previously (25). BN-PAGE gels were either used for native Western blotting (25) or used for second dimension separation or complexome profiling (26).

For separation in a second, denaturing dimension (two-dimensional BN/SDS-PAGE), lanes were cut and separated in Tricine-SDS-PAGE with 10% acrylamide. They were silver stained or used for Western blot as previously described (27).

##### Sample Preparation for Complexome Profiling

Blue native gels were fixed in 50% (v/v) methanol, 10% (v/v) acetic acid, 10 mm ammonium acetate for 30 min and stained with Coomassie (0.025% Serva Blue G, 10% (v/v) acetic acid) (25). Each lane was cut into 40 equal fractions and collected in 96-well filter plates (30–40 μm PP/PE, Pall Corporation). The gel pieces were destained in 60% methanol, 50 mm ammonium bicarbonate. Solutions were removed by centrifugation for 2 min at 600 × *g*. Proteins were reduced in 10 mm DTT, 50 mm ammonium bicarbonate for 1 h at 56 °C and alkylated for 45 min in 30 mm iodoacteamide. Samples were digested for 16 h with trypsin (sequencing grade, Promega) at 37 °C in 50 mm ammonium bicarbonate, 0.01% Protease Max (Promega), and 1 mm CaCl_2_. Peptides were eluted in 30% acetonitrile and 3% formic acid, centrifuged into a fresh 96-well plate, dried in a speed vacuum, and resolved in 1% acetonitrile and 0.5% formic acid (26).

##### Mass Spectrometry and Data Analysis of SILAC-based qCo-IP

Gel lanes of the combined light and heavy labeled samples were cut into 8 slices. SDS gel was fixed in 50% (v/v) methanol, stained with Coomassie Blue, and digested with trypsin as described in the complexome profiling sample preparation above.

Liquid chromatography/mass spectrometry (LC/MS) was performed on a Thermo Scientific^TM^ Q Exactive Plus coupled to an ultra-high performance liquid chromatography unit (Thermo Scientific Dionex Ultimate 3000) via a Nanospray Flex Ion Source (Thermo Scientific). Peptides were loaded on a C18 reversed-phase precolumn (Zorbax 300SB-C18, Agilent Technologies) followed by separation on an in-house packed 2.2-μm Reprosil Gold C18 resin (Dr. Maisch GmbH) picotip emitter tip (diameter 75 μm, 12 cm long, New Objectives) using a gradient from mobile phase A (4% acetonitrile, 0.1% formic acid) to 45% mobile phase B (80% acetonitrile, 0.1% formic acid) for 60 min with a flow rate of 250 nl/min. MS data were recorded by data-dependent Top10 acquisition (selecting the 10 most abundant precursor ions in positive mode for high energy collision dissociation fragmentation). The full MS scan range was 250 to 2,000 *m*/*z* with a resolution of 70,000 at *m*/*z* 200. Only higher charged ions (2+) were selected for MS/MS scans with a resolution of 17,500. MS data were analyzed by MaxQuant (version 1.5.2.8) (28) and searched against the human proteome database UniProtKB with 68,506 entries, released in 4/2015. The enzyme specificity was set to trypsin, missed cleavages were limited to 2 and the minimum peptide length was seven amino acids. The following variable modifications were selected: at N terminus acetylation (+42.01), oxidation of methionine (+15.99), deamidation on asparagine and glutamine (−0.98), and carbamidomethylation (+57.02) on cysteines. False discovery rate for protein and peptides was 1% (supplemental Table S1), which includes peptide and protein identification, accession numbers, protein and gene names, sequence coverage of each sample or gel slice, scores, and normalized SILAC ratios. Identifications from the reverse decoy database, by site and known contaminants were excluded. Further data analysis and scatter plots were generated by Perseus (version 1.5.1.6). Proteins with a log_2_ ratio >0.5 were considered as protein of interest. The mass spectrometry proteomics data have been deposited to the ProteomeXchange Consortium via the PRIDE partner repository (29) with the dataset identifier PXD003514.

##### Mass Spectrometry and Data Analysis for Complexome Profiling

LC/MS was performed on a Thermo Scientific Orbitrap XL mass spectrometer with an Agilent1200 nano-HPLC (high-performance liquid chromatography) at the front end. Peptides were loaded on a C18 reversed-phase precolumn (Zorbax 300SB-C18, Agilent Technologies) followed by separation on an in-house packed 3-μm Reprosil C18 resin (Dr. Maisch GmbH) picotip emitter tip (diameter 75 μm, 10 cm long, New Objectives) using a gradient from 5% acetonitrile, 0.1% formic acid to 50% acetonitrile, 0.1% formic acid for 90 min with a flow rate of 200 nl/min. MS data were recorded by data-dependent Top10 acquisition selecting the 10 most abundant precursor ions in positive mode for fragmentation using dynamic exclusion of 3 min with a resolution of 30,000 at 400 *m/z*. Only higher charged ions (2+) were selected for MS/MS scans in the linear ion trap by collision-induced dissociation at 42% collision energy. Lock mass option for *m*/*z* = 445.120025 (30) was enabled to ensure high mass accuracy during many following runs. MS data were analyzed by proteomics software Max Quant (1.5.2.8) (28) with the same settings as for qCo-IP. In addition, intensity-based absolute quantification values were recorded (supplemental Table S2). Abundance profiles were generated by NOVA software (0.5.7) (31) using intensity-based absolute quantification values from MaxQuant (32). Intensity-based absolute quantification values of proteins from the gel lane fraction were normalized to the maximum of the lane and hierarchically clustered using Pearson correlation distance function with average linkage and displayed as heat maps. The slice number of the maximum appearance of mitochondrial complex III dimer (483,695 Da), complex IV (210,786 Da), complex V (618,824 Da), and respiratory supercomplex containing complex I, III dimer, and one copy of complex IV (1,654,457 Da) was used for native mass calibration. The equation (*f*(*x*) = 8457 × *e*∧(0.1364*x*), *R*^2^ = 0.9934) obtained by exponential regression was used to calculate the native masses of each slice. The mass spectrometry proteomics data have been deposited to the ProteomeXchange Consortium via the PRIDE partner repository (29) with the dataset identifier PXD003509.

##### Western Blot Analysis

Triton or digitonin-lysed samples as well as differential centrifugation samples were substituted with sample buffer (8.5% glycerin, 2% SDS, 6.25% Tris/HCl, pH 6.8, 20 mm DTT, 0.013% bromphenol blue) but not heat denatured. As a reducing agent tris(2-carboxyethyl)phosphine (Thermo Scientific) was used as described (5). The protein amount was determined by Bradford protein assay. Proteins were separated by SDS-PAGE, substituted to Western blot, and detected by primary antibodies. Infrared fluorescent-labeled secondary antibodies were used and visualized in the Odyssey system (Licor, Bad Homburg, Germany).

NOX4 was detected by an anti-NOX4 antibody (1:2,000) reported by Anilkumar *et al.* (24). Primary antibody against NOX1 (Mox1, number sc-5821, 1:500), NOX5 (number sc-67006, 1:500), β-Tubulin (number sc-9104, 1:500), and HSPA5 (number sc-1051, 1:1,000) were purchased from Santa Cruz. Anti-calnexin (number MAB3126, 1:2,000) was obtained from Merck Millipore; Na/K-ATPase (number α6F, 1:2,000) from Hybridoma Bank, University of Iowa; Golgin (number A21270, 1:1,000) from Invitrogen; GAPDH (number G8795, 1:10,000) and β-Actin (number A1978, 1:2,000) from Sigma; and GFP from Roche (number 11814460001, 1:2,000). Antisera for mitochondrial proteins (ATP synthase subunit α or subunit β, Complex III) were used as described previously (26).

##### Proximity Ligation Analysis

Proximity ligation analysis (PLA) was performed according to the manufacturer's protocol (Duolink II Fluorescence, Duolink In Situ Detection Reagents Orange, OLink). Cells were fixed in 4% phosphate-buffered formaldehyde solution, permeabilized with 0.05% Triton X-100, blocked, and incubated overnight with rabbit monoclonal antibodies against NOX4 (provided by one of the co-authors (P.J.-D.)) and CANX antibody (Santa Cruz, number sc-6465) as well as negative and positive controls to validate the assay (data not shown). To show the specificity of the CANX antibody, a blocking peptide was used (Santa Cruz, number sc-6465 P). Samples were washed, incubated with the respective PLA probes for 1 h (37 °C), washed, and ligated for 30 min (37 °C). After an additional washing, amplification with polymerase was performed for 100 min (37 °C). The nuclei were stained with DAPI. Images were acquired by confocal microscope (LSM 510, Zeiss) and Plan-Neofluar ×40/1.3 oil objective at room temperature. Acquisition software Zen 2009 (Zeiss) was used. For each biological sample (*n* ≥ 3) eight pictures were evaluated and counted. Quantitative analysis was performed using Fiji software. Interactions were normalized to nuclei.

##### RNA Isolation and RT-qPCR

RNA was isolated by the RNA-Mini Kit (Bio&Sell) according to the manufacturer's instructions. Random hexamer primers (Promega) and SuperScript III Reverse Transcriptase (Invitrogen) were used for reverse transcription. The cDNA was used for semiquantitative real-time PCR using 5× QPCR MixEvaGreen® (Rox) (Bio&Sell) in the Mx3000P qPCR cycler (Agilent Technologies). Relative mRNA expression was normalized to β-Actin (β*Act*) or Polymerase IIa (*POLR2A*), as housekeeping gene, and analyzed by the ΔΔ*C_t_* method. Results are shown as relative to the corresponding control. Primer sequences are summarized in Table 1.

**TABLE 1:** **RT-qPCR primer**

Gene		Sequence
**h*POLRA***	Forward	5′-GCACCACGTCCAATGACAT-3′
	Reverse	5′-GTGCGGCTGCTTCCATAA-3′
**mβ*Act***	Forward	5′-AAAGACCTGTACGCCAACAC-3′
	Reverse	5′-GTCATACTCCTGCTTGCTGAT-3′
**h*NOX4***	Forward	5′-TCCGGAGCAATAAGCCAGTC-3′
	Reverse	5′-CCATTCGGATTTCCATGACAT-3′
**h*CANX***	Forward	5′-GGTGCTTGGAACTGCTATTG-3′
	Reverse	5′-CCCTGTTGGAACTGGAGCTT-3′

##### Statistical Analysis

Values are mean ± S.E. or S.D. as indicated. Statistical analysis was carried out using Prism 3.02. For multiple testing analysis of variance followed by post hoc testing (Bonferroni's multiple comparison test) was used. Unless otherwise indicated, *n* indicates the number of individual experiments. Densitometry was performed using Odyssey software (Image studio lite 5.0) and normalized to corresponding reference protein β-Actin. A *p* value of less than 0.05 was considered statistically significant (*, *p* < 0.05; **, *p* < 0.01; ***, *p* < 0.001).

## Results

### 

#### 

##### NOX4 Resides in Macromolecular Complexes

**FIGURE 1.**
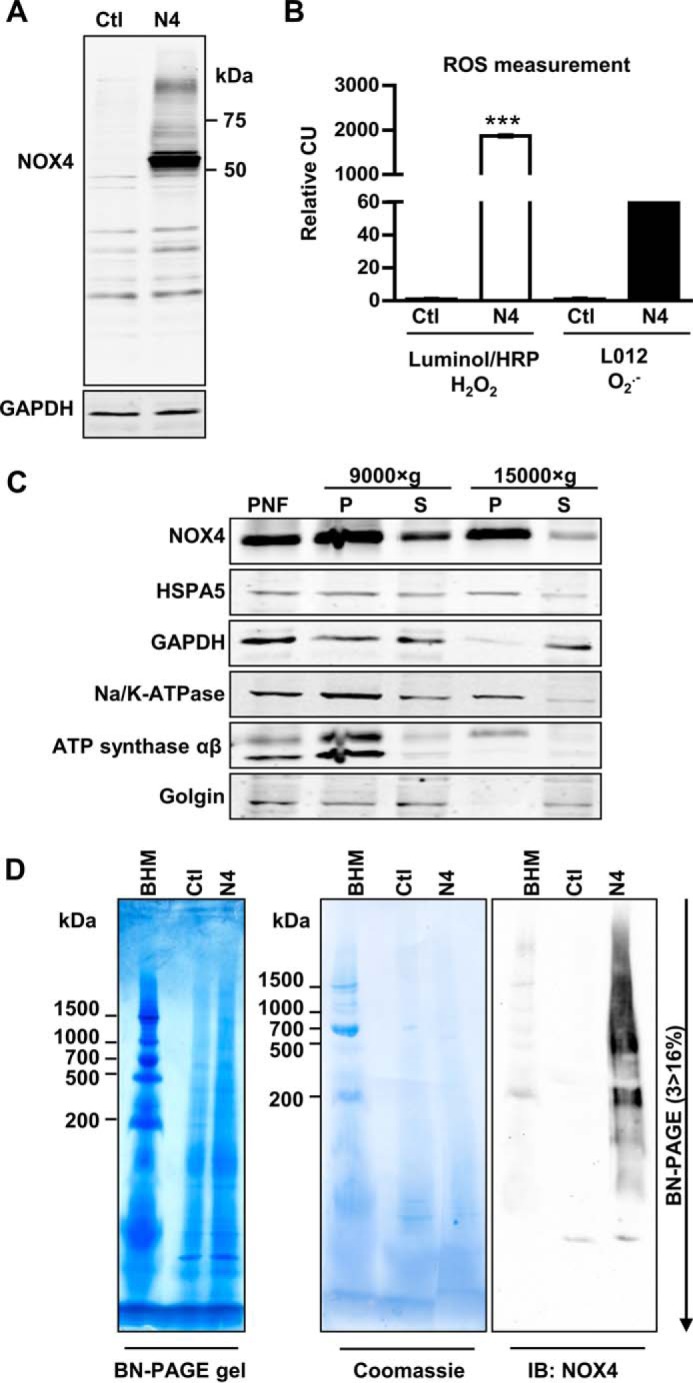
**Characterization, differential centrifugation, and BN-PAGE of HEK293 cells lentiviral transduced with NOX4.** *A*, representative Western blot for NOX4 in HEK293 cells (*Ctl*) and lentiviral-transduced NOX4-HEK293 cells (*N4*). *B*, luminol/HRP and L012 chemiluminescence assay in intact Ctl or N4-HEK293 cells normalized to Ctl cells (*CU*, chemiluminescence unit). *n* ≥ 3, mean ± S.D., *, *p* < 0.05; **, *p* < 0.01; ***, *p* < 0.001 relative to the corresponding Ctl HEK293 values. *C*, NOX4-HEK293 were mechanically homogenized and subcellular components were separated by differential centrifugation at 3,000 × *g* (*PNF*: postnuclear fraction), 9,000 × *g* and 15,000 × *g* to gain a pellet (*P*) and supernatant (*S*). *D*, 15,000 × *g* membrane pellets were solubilized with digitonin (6 g/g of protein) and used for BN-PAGE (3 > 16% acrylic amide); bovine heart mitochondria (*BHM*) served as weight standard. BN-PAGE gels were native blotted on a PVDF membrane and subsequently stained with Coomassie Blue and immunoblotted with anti-NOX4 antibody.

**FIGURE 2.**
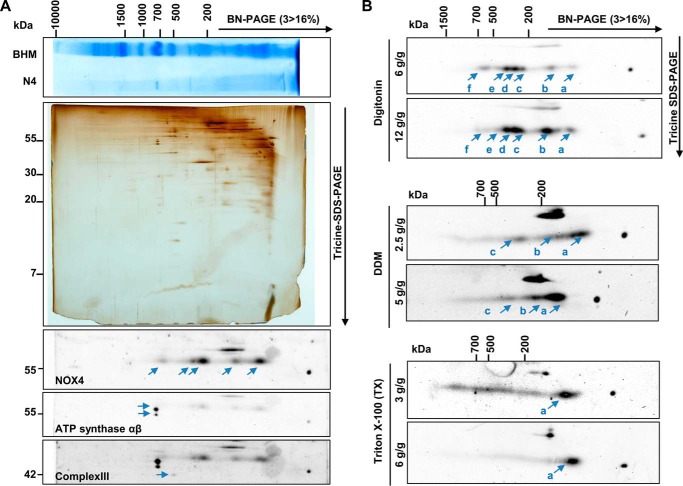
**Multidimensional electrophoretic resolution of NOX4-HEK293 membranes by two-dimensional BN/SDS-PAGE.** *A*, 15,000 × *g* membrane pellet from differential centrifugation of NOX4-HEK293 (N4) cells was solubilized with digitonin (6 g/g of protein) and separated by one-dimensional BN-PAGE (3 > 16% acrylamide) with subsequent two-dimensional separation by Tricine-SDS-PAGE with 10% acrylamide. Representative BN-PAGE gel and silver staining of the two-dimensional BN/SDS-PAGE gel are shown. The two-dimensional BN/SDS-PAGE gel was blotted and sequential antibody detection was performed. Protein complexes of the mitochondria (bovine heart mitochondria; *BHM*) were used as size standard. *B*, representative two-dimensional BN/SDS-PAGE blots of NOX4 solubilized with different detergents (digitonin, *DDM* (*n*-dodecyl β-d-maltopyranoside), or Triton X-100) and concentrations as indicated.

As endogenous NOX4 is expressed to a low level only, we used an overexpression system to find NOX4-interacting proteins. Human NOX4 was stably overexpressed in HEK293 (N4) cells. The cells were characterized by a highly abundant expression of NOX4 and a constitutive production of reactive oxygen species (Fig. 1, *A* and *B*). To reduce background proteins and complexity of the homogenate, NOX4 could be enriched in the 15,000 × *g* pellet after differential centrifugation and used for subsequent analysis (Fig. 1*C*). To test the hypothesis of NOX4 interacting proteins, BN-PAGE (Fig. 1*D*) followed either by native Western blotting of the one-dimensional BN-PAGE (Fig. 1*D*) or by two-dimensional SDS-PAGE and Western blot analysis (Fig. 2) was performed from membrane extracts of HEK293 cells stably transduced with NOX4. This technique revealed that besides the NOX4 monomer, higher molecular weight complexes are present in HEK293 cells. One-dimensional BN-PAGE Western blot demonstrated Nox4 complexes up to over 1500 kDa (Fig. 1*D*). NOX4 complexes could be further separated in a denaturating two-dimensional SDS-PAGE to detect complexes of ∼67, 117, 247, 286, 358, and 580 kDa (Fig. 2*A*). To better define the stability and the nature of the complexes, detergents and their concentrations were varied. The complexes were detected after membrane solubilization with digitonin and also to some extent in maltopyranoside-lysed membranes. In contrast, after Triton lysis, a single dot with a mass of ∼83 kDa was retrieved (Fig. 2*B*). Thus, NOX4 resides in macromolecular complexes in HEK293 cells overexpressing the protein.

##### Calnexin Co-immunoprecipitates with NOX4

**FIGURE 3.**
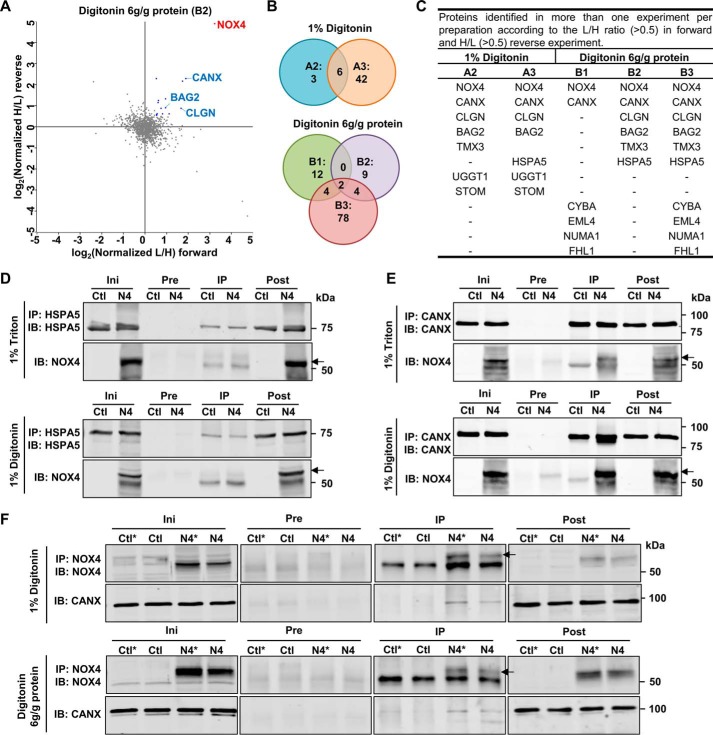
**Determination of interaction partners using qCo-IP and standard Co-IP with subsequent Western blot analysis.** HEK293 (*Ctl*) and NOX4-HEK293 (*N4*) cells were cultured in normal or heavy labeled (*) SILAC medium (^13^C-R; ^13^C^15^N-K), lysed with either 1% digitonin (*n* = 2) in TBS or 100,000 × *g* total membrane pellets (*n* = 3) were solubilized with digitonin (6 g/g of protein). Samples were used for NOX4 qCo-IP with magnetic beads and subsequent MS detection. *A*, logarithmic normalized L/H ratios of forward (N4/Ctl*) and H/L in reverse (N4*/Ctl) experiment analyzed by MS were exemplary plotted against each other (experiment *B2*). Background proteins show a log_2_(H/L) ratio around 0 in both experiments, specific interactors have a high log_2_(L/H) in the forward and a high log_2_(H/L) ratio in the reverse experiment. NOX4 is highlighted in *red* and proteins with a threshold over 0.5 in *blue*. Proteins of interest are labeled *B*, Venn diagram compares proteins found in different experiments with a threshold >0.5 (*n* = 2; *n* = 3). *C*, table showing proteins with threshold of >0.5 found in more than one experiment per preparation. *D* and *E*, representative Western blot for NOX4 in initial (*Ini*), preclearing (*Pre*), immunoprecipitation (*IP*), and post-IP fraction after lysis with 1% Triton -X-100 or digitonin of HEK293 (*Ctl*) and NOX4-HEK293 (*N4*). Co-IP was performed with anti-HSPA5 (*D*) or anti-CANX (*E*) antibody and Sepharose beads, *n* ≥ 3. *F*, representative Western blot of CANX in Ini, Pre, IP, and post-IP fraction after Co-IP with NOX4 antibody. Ctl and N4 cells (used for SILAC-based Co-IP), lysed with either 1% digitonin in TBS (*n* = 2) or 100,000 × *g* membrane pellets were solubilized with digitonin (6 g/g of protein; *n* = 3). *IB*, immunoblot.

To identify NOX4 interacting proteins, the strategy of the qCo-IP/mass spectrometry approach (33) was carried out in HEK293 cells, with and without stable NOX4 overexpression and lysed with different concentrations of digitonin and ionic strengths (Fig. 3 and supplemental Table S1). Also experiments with endogenous NOX4 were performed but its expression level was too low to recover sufficient amounts of NOX4 (data not shown). On this basis it was concluded that overexpression was needed for these experiments. Once overexpressed, NOX4 could be immunoprecipitated in all experiments and several other proteins were specifically enriched in the precipitate of NOX4 overexpressing cells (Fig. 3, *A–C*). NOX4 was a little higher expressed in the cell line grown in heavy SILAC medium (*) and therefore reverse values exceed forward experiments, which set a small bias for the numeric analysis (Fig. 3, *A* and *F*). Nevertheless, NOX4 could be recovered in all five experiments with reliable values far over the threshold of 0.5 in forward (log_2_(normalized L/H); NOX4/Ctl*) and reverse (log_2_(normalized H/L); NOX4*/Ctl) experiments. In all five independent experiments each comparing forward and reverse experiments, calnexin was specifically co-precipitated, whereas others, like the 78-kDa glucose-regulated protein (HSPA5/Grp78) were only identified by MS in some isolates (Fig. 3*C*). Described known interacting proteins like p22*^phox^*/CYBA or SERCA2b/ATP2A2 (sarco/endoplasmic reticulum Ca^2+^-ATPase 2b) were detected in one or two experiments by this approach. To verify the MS data, Western blot analysis was carried out (Fig. 3, *D–F*). With this technique, anti-calnexin antibodies co-precipitated NOX4 (Fig. 3*E*) and anti-NOX4 antibodies co-precipitated calnexin (Fig. 3*F*). The interaction was more robust after digitonin lysis but also detectable in 1% Triton lysis buffer. Despite the unspecific signal of the antibody used for the immunoprecipitation (50 kDa) HSPA5 did not efficiently coprecipitate NOX4 in either of the conditions (Fig. 3*D*).

##### Calnexin and NOX4 Reside in the Same Complexes

**FIGURE 4.**
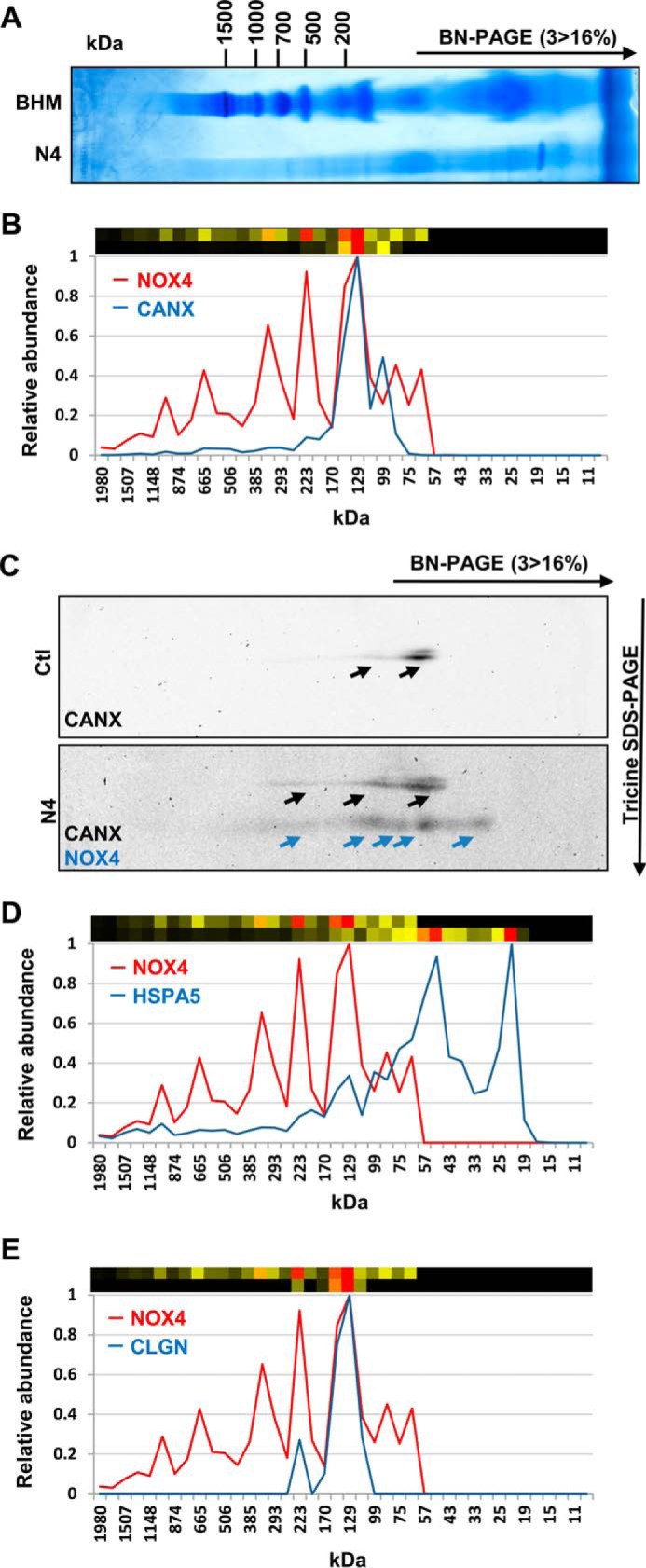
**Migration profiles of NOX4 and Calnexin, HSPA5, or calmegin, identified by complexome profiling and two-dimensional BN/SDS-PAGE.** One-dimensional BN-PAGE of NOX4 15,000 × *g* membrane pellets (6 g of digitonin/g of protein) (*A*) was analyzed by MS-based Complexome profiling (*B*, *D*, and *E*). Relative abundance of MS identified NOX4 (*red*) and CANX (*B*) HSPA5 (*D*) and calmegin (CLGN) (*E*) (*blue*) are plotted against the apparent molecular mass separated by one-dimensional BN-PAGE. Corresponding heat maps are shown *above* (*upper*, NOX4; *lower*, CANX, HSPA5, and CLGN). *Red* indicates the highest intensity and *black* the lowest intensity. *C*, representative two-dimensional BN/SDS-PAGE Western blot of membrane preparation of Ctl and NOX4-HEK293 cells and immunoblotting with CANX and NOX4 as indicated.

To study whether calnexin is a member of the NOX4 macromolecular complex, samples from BN-PAGE, originated from the NOX4-enriched 15,000 × *g* pellet of the NOX4-HEK cells (Figs. 1 and 4*A*), were subjected to complexome profiling (supplemental Table S2) (26). NOX4 and calnexin were both specifically present in a complex of 100–170 kDa, but some NOX4 proteins also co-migrate in complexes up to 1,000 kDa with calnexin (Fig. 4, *B* and *C*). Obviously, given the complex procedure from the membrane preparation, resolution by large gradient gels and electrophoresis in a second dimension, BN-PAGE has some variability as also seen in the present study. Nevertheless, as also detected by Western blot from the second dimension, the migration pattern of calnexin was very similar to that of NOX4 (Fig. 4*C*).

Interestingly, other potential NOX4-interacting proteins retrieved by the SILAC-based approach also exhibited a presence in specific complexes, but only calmegin (CLGN) and HSPA5 showed a migration profile similar to that of calnexin (Fig. 4, *D* and *E*). The interaction with NOX4 of these proteins, however, appears to be less strong than that of calnexin. By Co-IP followed by Western blot, we failed to co-precipitate significant amounts of NOX4 with HSPA5 (Fig. 3*D*).

##### The Interaction with Calnexin Is Specific for NOX4

**FIGURE 5.**
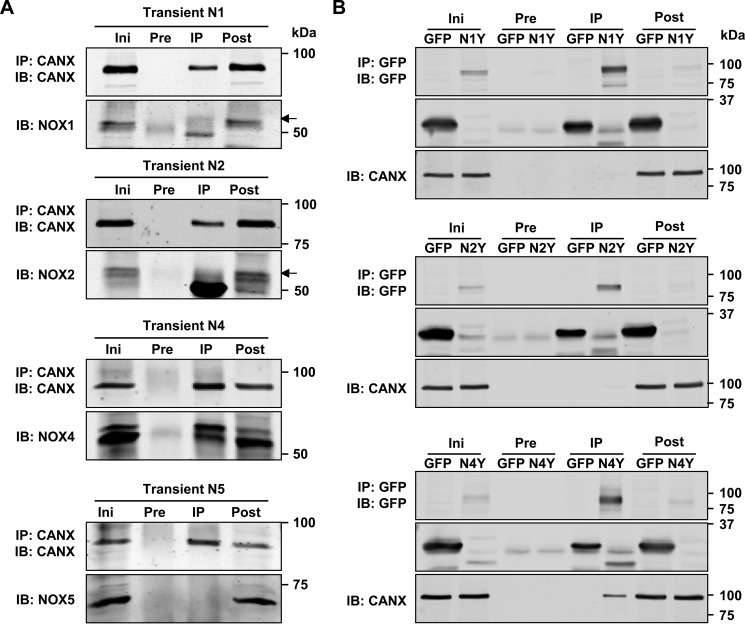
**CANX and NOX1, -2, -4, and -5 interaction in Co-IP.** *A*, HEK293 cells were transiently transfected with human NOX1, NOX2, NOX4, and NOX5. Representative Western blot for NOX1, -2, -4, and -5 in initial (*Ini*), preclearing (*Pre*), immunoprecipitation (*IP*), and post-IP fraction after Co-IP with anti-CANX. *B*, HEK293 cells were transiently transfected with GFP as control and NOX1-YFP (*N1Y*), NOX2-YFP (*N2Y*), and NOX4-YFP (*N4Y*). Representative Western blot for transfected constructs and co-immunoprecipitated CANX in Ini, Pre, IP, and post fraction after Co-IP with anti-GFP antibody. *IB*, immunoblot.

To address whether the interaction of calnexin is specific for NOX4, co-immunoprecipitation experiments were performed in HEK293 cells transiently overexpressing NOX4 or other NOX homologues as well as previously generated YFP-NOX (yellow fluorescent protein) fusion proteins (16) to overcome background and antibody problems. Also after transient overexpression, NOX4 and calnexin could be co-precipitated (Fig. 5*A*) and this interaction could be confirmed for the NOX4-YFP fusion construct with ∼90 kDa. GFP alone (25 kDa) did not co-immunoprecipitate calnexin (Fig. 5*B*). In contrast, NOX1, NOX2, or NOX5 did not exhibit this behavior (Fig. 5*A*), even if fused to YFP (Fig. 5*B*, approximately 90 kDa). Thus, the interaction with calnexin is specific for NOX4.

##### Calnexin Is Required for NOX4 Protein Expression in Stably Transduced HEK293 Cells

**FIGURE 6.**
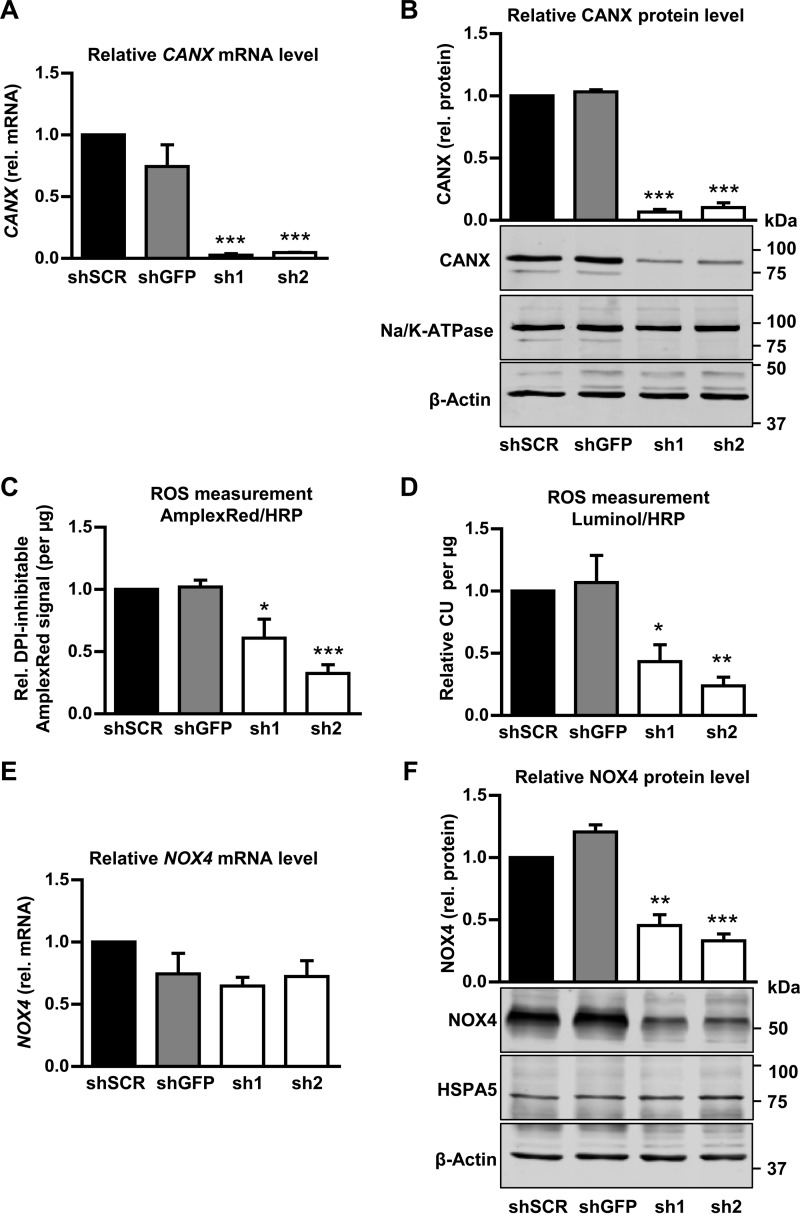
**NOX4-dependent H_2_O_2_ production in shCANX-treated NOX4-HEK293 cells.** *A* and *E*, normalized, relative mRNA level for *CANX* and *NOX4* in controls (scrambled shRNA: *shSCR, shGFP*) and shCANX-treated NOX4-HEK293 cells (*sh1* and *sh2*). *B* and *F,* representative Western blot with densitometry for NOX4 and CANX protein expression normalized to β-Actin and shSCR in controls (shSCR and *shGFP*) and after shRNA-mediated CANX knockdown (*sh1* and *sh2*). *C*, relative diphenylene iodonium-sensitive (10 μm) Amplex Red/HRP assay of controls (*shSCR* and *shGFP*) and after shRNA-mediated CANX knockdown (*sh1* and *sh2*) normalized to protein amount (μg). *D*, relative luminol/HRP assay in controls (*shSCR* and *shGFP*) and after shRNA-mediated CANX knockdown (*sh1* and *sh2*). 100,000 cells per measurement were used and CU (chemiluminescence unit) was normalized to protein amount (μg) and *shSCR* control. For all ROS measurements, three independent measurements were performed for each of the three biological replicates. Also shown are representative Western blots for Na/K-ATPase (*B*) and HSPA5 (*F*) protein expression in controls (*shSCR* and *shGFP*) and after shRNA-mediated CANX knockdown (*sh1* and *sh2*), *n* ≥ 3, mean ± S.E. *, *p* < 0.05; **, *p* < 0.01; ***, *p* < 0.001 relative to the corresponding shSCR-treated cells.

Calnexin is an endoplasmic reticulum chaperone. For a functional significant interaction of NOX4 with calnexin either NOX4 has to control the redox state of calnexin or vice versa, calnexin has to be involved in the protein folding and maturation of NOX4. Given that little evidence so far has suggested that NOX4 controls the redox state of oxidative protein folding in the ER (34), the second aspect was analyzed by a loss of function approach with the aid of calnexin shRNA. Two different shRNAs were designed that reliably down-regulated calnexin mRNA and protein (Fig. 6, *A* and *B*). Loss of calnexin decreased the H_2_O_2_ production of NOX4-overexpressing HEK293 cells (Fig. 6, *C* and *D*). Importantly, calnexin shRNA decreased the protein level of Nox4, whereas *NOX4* mRNA remained unchanged (Fig. 6, *E* and *F*). Of note, calnexin was not required to maintain levels of all proteins generated in the ER as the expression of Na/K-ATPase was not affected by calnexin shRNA (Fig. 6*B*). Moreover, HSPA5, another important ER chaperone, which was also recovered in some of the SILAC/MS-based co-immunoprecipitation experiments, was not required to maintain the NOX4 protein level and ROS production (Fig. 6*F*). Thus, calnexin is required to maintain proper levels of NOX4.

##### Calnexin Interacts with the Physiologically Expressed Endogenous NOX4

**FIGURE 7.**
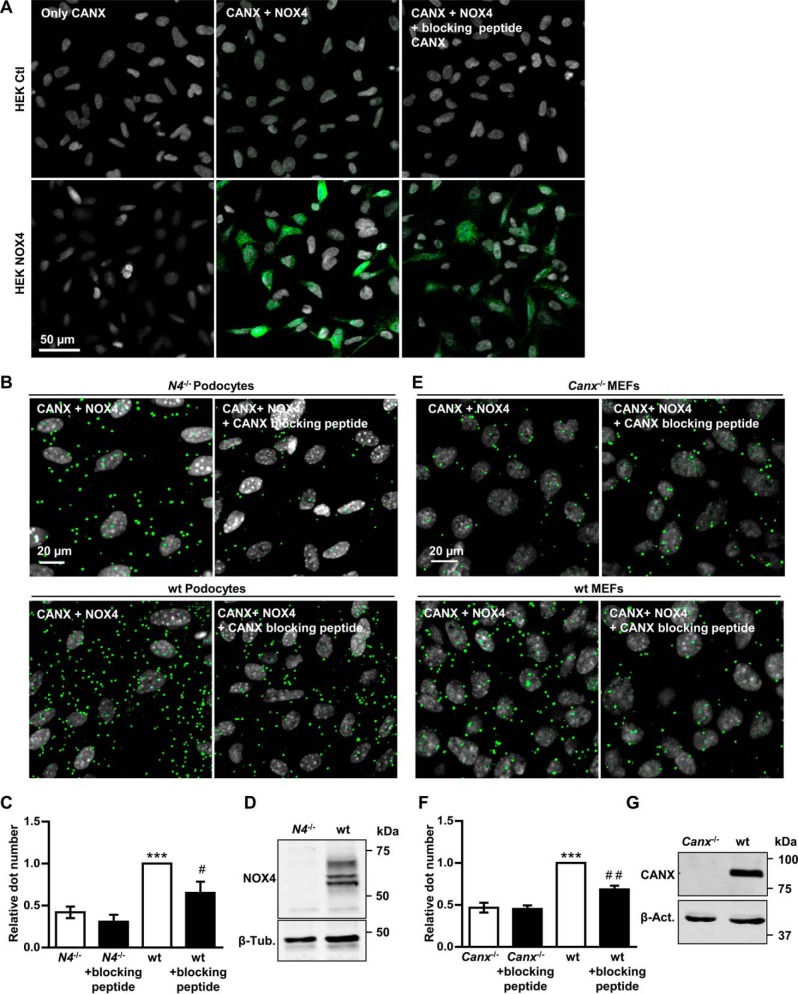
**CANX and NOX4 interaction in proximity ligation assay analysis.** *A*, representative images of proximity ligation assay analysis showing the interaction of NOX4 with CANX in HEK293 cells with (*NOX4*) and without (*Ctl*) overexpression of NOX4. Positive signal of interaction is shown as *green dots*, nuclei are stained with DAPI (*gray*). In the *two left images* the primary antibody against NOX4 was omitted, in the *right images*, a blocking peptide against calnexin was added. *B* and *E*, representative images of proximity ligation assay analysis showing the interaction of NOX4 with CANX in podocytes from wt or *Nox4*^−/−^ (*N4*^−/−^) mice (*B*) or MEF from wt or *Canx*^−/−^ mice (*E*). *C* and *F*, dots per nuclei were analyzed of at least three independent experiments (8 pictures each) and normalized to wt cells. CANX antibody blocking peptide was used as control, *n* ≥ 3, mean ± S.E., *, *p* < 0.05; **, *p* < 0.01; ***, *p* < 0.001 relative to the corresponding knock out; #, *p* < 0.05, ##, *p* < 0.01 relative to the control without blocking peptide D. *G*, representative Western blot characterizing the *Nox4*-deficient podocytes (*D*) or *Canx*-deficient MEFs (*E*). As loading control β-Tubulin (*C*, β-*Tub*.) or β-Actin (*G*, β-*Act*.) was used.

Stable overexpressing HEK293 cells are a frequently used model in NOX research but have strong limitations. They yield no information about endogenous NOX expression, function, and activity and aberrant expression and high turnover of the target protein could yield false-positive interactions. On this basis, an attempt was made to demonstrate that endogenous NOX4 interacts with calnexin. Unfortunately, we consistently failed to enrich endogenous NOX4 sufficiently by immunoprecipitation to perform MS qCo-IP experiments. As an alternative, PLA were performed. This technique was first validated in HEK293 cells overexpressing NOX4. By PLA, these cells yielded a strong interaction signal, which was largely attenuated by a blocking peptide against the calnexin antibody. Native HEK293 cells, which express very little NOX4, in contrast, were PLA negative for the interaction of NOX4 and calnexin (Fig. 7*A*). Next, murine podocytes and fibroblasts were studied as these cells express NOX4 more abundantly than other cells. By PLA, the interaction of endogenous NOX4 with calnexin could indeed be demonstrated in murine podocytes as well as in MEFs (Fig. 7, *B–G*). Blocking peptides for the anti-calnexin antibody significantly attenuated the interaction signal. Moreover and importantly, genetic deletion of either *Nox4* (Fig. 7, *B–D*) or calnexin (Fig. 7, *E–G*) was equally effective as the blocking peptides in reducing the interaction signal. The overall signal intensity in the experiments focusing on endogenous NOX4 was much lower than after overexpressing (35) the protein and required more amplification. Also the signal to background ratio was lower as the antibodies used were not made for immunofluorescence and as murine cells compared with human cells yield a different unspecific reaction pattern. Despite this, it can be concluded that endogenous NOX4 interacts with calnexin in MEFs and podocytes.

##### Genetic Deletion of Calnexin Affects NOX4 Expression

**FIGURE 8.**
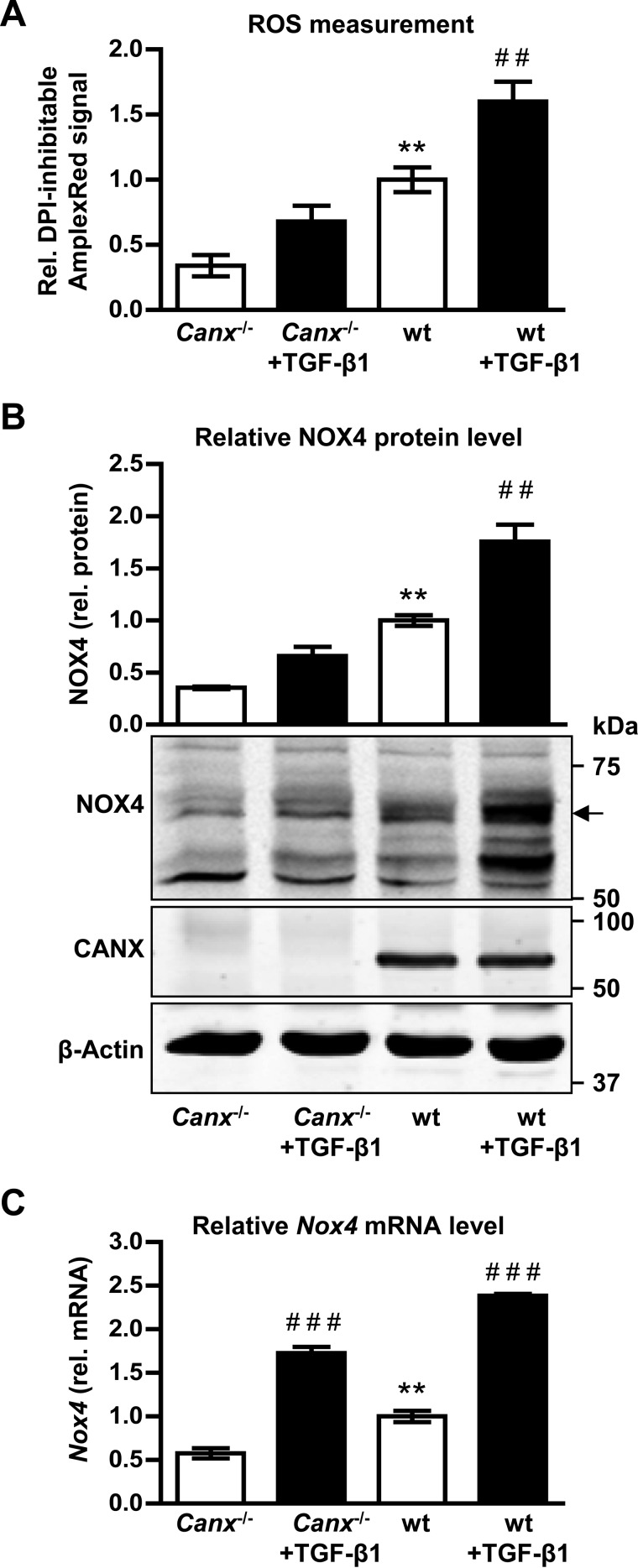
**NOX4-dependent H_2_O_2_ production in *Canx*-deficient MEF.** *A*, relative diphenylene iodonium (*DPI*; 10 μm, 1 h) sensitive Amplex Red/HRP signal in *Canx*^−/−^ or wt MEFs after TGF-β1 treatment (10 ng/ml, 24 h). *B*, representative Western blot and densitometry of NOX4 (highlighted upper NOX4 band) and CANX protein expression normalized to β-Actin and wt control in *Canx*^−/−^ or wt MEFs. *C*, normalized, relative mRNA level of *Nox4* mRNA in *Canx*^−/−^ or wt MEFs. *n* ≥ 3, mean ± S.E. *, *p* < 0.05; **, *p* < 0.01; ***, *p* < 0.001 relative to the corresponding wt cells; #, *p* < 0.05; ##, *p* < 0.01; ###, *p* < 0.001 relative to the corresponding non-treated cells.

To determine whether calnexin is also of relevance for endogenously expressed NOX4, experiments were carried out in MEFs. As compared with wild type (wt) MEFs, H_2_O_2_ production was significantly lower in calnexin^−/−^ MEFs. Stimulation of cells with TGF-β1 (10 ng/ml), a known inducer of NOX4 for 24 h increased ROS production in wt MEFs. This effect was smaller in calnexin^−/−^ MEFs (Fig. 8*A*). Similar to NOX4 overexpressing HEK293 cells, genetic deletion of calnexin was associated with reduced protein levels of NOX4 under basal conditions as well as after stimulation with TGF-β1 (Fig. 8*B*). In contrast to the observations in HEK293 cells, *Nox4* mRNA expression was also lower in calnexin^−/−^ MEFs as compared with wt MEFs. TGF-β1 resulted in a similar increase in mRNA expression between the two cellular systems (Fig. 8*C*), whereas NOX4 protein accumulation in response to this cytokine was greatly reduced in calnexin^−/−^ cells as compared with the wt control cells (Fig. 8*B*). Thus, the endogenous protein levels of NOX4 are controlled by calnexin in MEFs.

## Discussion

In the present study we identified the ER chaperone calnexin as a novel NOX4 interacting protein. Calnexin specifically co-precipitated with overexpressed NOX4 and PLA also demonstrated interaction of native NOX4 with calnexin. Functionally, loss of calnexin attenuated NOX4-dependent H_2_O_2_ formation in cultured cells. Thus, this work establishes that in addition to expression control, protein interaction also impacts on NOX4-dependent ROS production.

Calnexin is a highly abundant ER protein, which has multiple functions. It is frequently used as an ER marker and its interaction with NOX4 illustrates that NOX4 resides in the ER. The ER location, however, was to some extent to be expected as integrative membrane proteins like NOX4 are usually synthesized by ribosomes located at the ER membrane. Whether from there NOX4 is subsequently transferred to other sites was beyond the scope of this work, but at least in a previous study in HEK293 cells we could exclude the plasma membrane as an important site of NOX4 expression (10). Moreover, the classic plasma membrane-bound NOX enzymes NOX2 and NOX1 are all synthesized into the ER and only after maturation does NOX2 exit this structure (15). Nevertheless, we failed to detect a significant interaction of NOX2 with calnexin. This suggests that the interaction of NOX4 and calnexin extends beyond what is usually required for protein maturation and that there is a constitutive interaction of NOX4 with the protein. Why this interaction is needed to maintain NOX4 protein levels is unclear. NOX4, unlike the other NOX homologues, tends to aggregate and thus increased folding activity might be needed to maintain it in its native state. Indeed, as compared with NOX2, NOX4 protein stability is scored lower by the instability index II predicted by the ExPASy ProtParam tool (NOX2: 45.77, NOX4: 34.69) (36).A potential analogy of NOX4/calnexin heterocomplex function can be seen in the cytoplasmic chaperone complexes with other NOX proteins. For example, Hsp70 or Hsp90 are important chaperones in this compartment and their role in the maintenance of protein function and not just protein folding is known. For example, the steroid hormone/hydrocarbon receptors require these chaperones to maintain a conformation that favors ligand binding and activation. Interestingly Hsp90 binds to the C terminus of NOX1, -2, -3, and NOX5, but not NOX4, to control superoxide formation and protein expression. An analog of cytoplasmic Hsp90 calnexin could substitute for the chaperone function of NOX4 in the ER and maintain activity and protein expression. Interaction studies in cell lysates always bear the potential of unspecific interactions occurring after cell lysis. Given the fairly high abundance of calnexin and NOX4, if overexpressed, this aspect is of potential concern. Several lines of evidence, however, speak against an artificial interaction occurring *ex vivo*. First, NOX4, even if not overexpressed, and calnexin also interacted *in vivo*, as demonstrated by the PLA assay, in which the cells are not disrupted. Moreover, although HSPA5 is as common in the ER as calnexin, we could exclude a firm interaction of NOX4 with this chaperone. Finally, we provide functional evidence for a significant interaction of NOX4 and calnexin as altering calnexin expression by shRNA or genetic knock-out also changes NOX4 protein expression and activity.

In addition to the interaction with calnexin, several other proteins were identified as potential interaction partners and some were not localized in the ER. Given the inconsistent recovery by the MS we did not further study these proteins and thus these interactions could potentially represent false-positives. This consideration also applies for the complexome analysis. As these experiments are performed in crude membrane preparations, proteins naturally residing in different compartments can aggregate during the preparation procedure. Unfortunately, we failed to prepare sufficient amounts of pure ER membrane proteins to perform the BN-PAGE, which is the basis for complex analysis. Obviously, the problem does not occur of source tissue is available in great abundance and if the preparation protocols yield a more pure organelle extract, as in the case of bovine heart mitochondria.

It was unexpected that p22*^phox^* was only retrieved twice in five SILAC qCo-IP experiments, despite the fact that p22*^phox^* has previously been established as an interacting protein of NOX4 (16, 17). p22*^phox^* is, however, difficult to detect by MS. It is a small transmembrane protein and cleavage by trypsin theoretically yields nine peptides, big enough for MS detection (>7 amino acids). Only four are small enough for efficient recovery from gel pieces. The larger peptides could escape identification due to less efficient elution from acrylamide gels. Moreover, by focusing on the ER, all stages of NOX4 maturation were recovered in the present study and it is unknown at what stage of protein folding the interaction with p22*^phox^* occurs. From leukocyte NOX2, it is known that the p22*^phox^* interaction is required for trafficking to the plasma membrane and that loss of p22*^phox^* also results in absence of NOX2 in leukocytes and vice versa (15, 37). This model, however, cannot be applied to NOX4, as membrane trafficking is not established for this enzyme. Thus, it is conceivable that a substantial portion of NOX4 in the ER resides in a p22*^phox^*-unbound form. A specific assay for NOX4 would, however, be required to further address this aspect by reconstitution experiments. Recently, a membrane assay of NOX4 after FPLC preparation of the enzyme has been published (38). Unfortunately, we failed to reconstitute NOX4 activity as published in that particular work. As an alternative, the lucigenin chemiluminescence assay is frequently advocated as a tool to analyze NOX activity in isolated membranes. We (23) and others (39), however, found no evidence that this assay detects NOX4 activity. In fact, overexpression of NOX4 had absolutely no effect on the NADPH-stimulated chemiluminescence signal of HEK293 cells (23). This situation is unfortunate as several other important questions remain, *i.e.* whether aggregate formation of NOX4 occurs and how much of the ROS signal generated by NOX4 derives from other sources, like mitochondria (40). In fact, given the tendency of NOX4 to precipitate, ER stress and pro-apoptotic signals can be a consequence of NOX4 overexpression, which would result in ROS formation unrelated to NOX4 (41, 42).

Calnexin as a type I ER membrane protein prolongs the retention time of misfolded proteins in the ER. It is therefore involved in quality control of the secretory pathway. Together with the oxidoreductase ERp57, calnexin is also responsible for proper folding of newly synthesized proteins (43). Moreover, calnexin similar to calreticulin is thought to act as a chaperone for glycoproteins (44). With respect to the interaction with NOX4, this feature is unexpected as so far no evidence has been presented that NOX4 is glycosylated. This aspect might be different to NOX2, which at least in the human form runs as a 91-kDa smear on the gel as a consequence of glycosylation (45). Although the particular running behavior of NOX2 in gels has been a prominent feature of the protein, glycosylation of NOX2 appears unimportant for its function (46). On SDS-PAGE, NOX4 also runs on a second band with a higher molecular mass than the one calculated from the amino acid sequence. Nevertheless, the glycosylation sites of NOX2 are not conserved in NOX4 and *N*-glycosidase F treatment did not reduce the NOX4 to a single band (47). Therefore the nature of the post-translation modification of NOX4 is unknown but ubiquitination has been suggested as a possible mechanism (48). It should, however, be noted that the calnexin-ERp57 complex selectively interacts with mono-glycosylated proteins and therefore this low degree of modification might very well be present in NOX4 and not yet recognized. Nevertheless, also binding of NOX4 by calnexin in a glycan-independent manner could be possible as previously described for other membrane proteins in the ER (49, 50).

Calnexin also binds other ER chaperones, in particular ERp57 (51) a protein-disulfide isomerase isoform. Interestingly, protein-disulfide isomerases are not only essential for ER protein folding; they are also implicated in NOX function. Knockdown of the main ER protein-disulfide isomerase reduced ROS formation by NOX enzymes in smooth muscle cells (19) but also in macrophages (52).

A second function of the complex of calnexin and NOX4 might be a contribution to ER homeostasis (53, 54). Both proteins co-localize with SERCA (55) and the ryanodine receptor (56) and deletion of NOX4 reduces cytosolic calcium levels. This aspect might be very relevant as protein folding requires oxygen and competes with mitochondria for this important substrate. Indeed, NOX4 has been discussed as a redox-sensor coupling oxygen tension to ROS formation (38) and therefore oxygen to intracellular calcium homeostasis (53, 57). Conceptually, this aspect may help to understand why NOX4 produces ROS in the ER but itself is not involved in oxidative protein folding, which is mediated by ERO-1 (34). Thus, although NOX4 may change the oxidation state of peroxiredoxin IV (58), this function might be of utility for signaling but not for folding *per se*. This concept is in line with previously published redox targets of NOX4, which are related to the ER: PTP1B (59) and SERCA2b (55).

In conclusion, with the present study we have identified calnexin as a novel NOX4 interacting protein. The interaction was required to maintain NOX4 protein level and activity. Changing calnexin abundance might therefore be a novel strategy to change NOX4-depedent ROS production.

## Author Contributions

K. K. P., I. W., M. S. L., J. G., N. W., M. M., P. J. D., A. M. J., and R. P. B. designed experiments, provided tools, critical discussions, and comments. K. K. P. and I. W. performed experiments. K. K. P., I. W., and R. P. B. analyzed data. K. K. P. and R. P. B. reviewed the data and wrote the manuscript.

## Supplementary Material

Supplemental Data

## Footnotes

4 The abbreviations used are:

ROS: reactive oxygen species
NOX4: NADPH oxidase 4
BN-PAGE: blue native-PAGE
SILAC: stable isotope labeling of amino acids in cell culture
(q)Co-IP: quantitative SILAC-based co-immunoprecipitation
MEF: mouse embryonic fibroblasts
H: heavy SILAC ([^13^C]arginine; [^13^C,^15^N]lysine)
L: light SILAC ([^12^C,^14^N]arginine
[^12^C,^14^N]lysine);PLA: proximity ligation analysis
Tricine: *N*-[2-hydroxy-1,1-bis(hydroxymethyl)ethyl]glycine
ER: endoplasmic reticulum.
