# Supplementary material for: The Endoplasmic Reticulum Chaperone Calnexin Is a NADPH Oxidase NOX4 Interacting Protein
Source: J Biol Chem. 2016 Feb 9;291(13):7045–59. doi: 10.1074/jbc.M115.710772 (PMC4807287; doi:10.1074/jbc.M115.710772)
Supplement: Supplemental Data [file supp_291_13_7045__index.html]

The endoplasmic reticulum chaperone calnexin is a NADPH oxidase Nox4 interacting protein — The Endoplasmic Reticulum Chaperone Calnexin Is a NADPH Oxidase NOX4 Interacting Protein — NOX4 Interacts with Calnexin — Supplemental Data 

# The Endoplasmic Reticulum Chaperone Calnexin Is a NADPH Oxidase NOX4 Interacting Protein

## Supplemental Data

- Supplemental table 1 (.xlsx, 1.8 MB) - Suppl. table 1 SILAC based quantification of proteins to identify interacting proteins with Nox4
- Supplemental table 2 (.xlsx, 641 KB) - Suppl. table 2 Complexome profiling of membrane proteins of Nox4-HEK293 cells
